# Cardiac tamponade during venoarterial extracorporeal membrane oxygenation: a case report

**DOI:** 10.1186/s13256-022-03741-9

**Published:** 2023-02-09

**Authors:** E. J. M. Adriaansen, J. A. J. Hermens, M. Broome, L. Pladet, E. Dubois, D. W. Donker, C. L. Meuwese

**Affiliations:** 1 Department of Intensive Care, Utrecht University Medical Center, Utrecht, The Netherlands; 2 ECMO Department, Karolinska University Hospital, Stockholm, Sweden; 3 Division of Anesthesia and Intensive Care, Department of Physiology and Pharmacology, Karolinska University Hospital, Stockholm, Sweden; 4 Department of Intensive Care, Erasmus Medical Center, Rotterdam, The Netherlands; 5 Department of Cardiology, Erasmus Medical Center, Rotterdam, The Netherlands; 6 Cardiovascular and Respiratory Physiology Group, TechMed Centre, Faculty of Science and Technology, University of Twente, Enschede, The Netherlands

**Keywords:** Venoarterial extracorporeal membrane oxygenation, Cardiac tamponade, Pericardial effusion, Weaning failure, Case report

## Abstract

**Background:**

Cardiac tamponade may present with very different signs and clinical consequences in patients who are supported with venoarterial extracorporeal membrane oxygenation. Failure to recognize cardiac tamponade in this setting can cause failure to wean from venoarterial extracorporeal membrane oxygenation, and even lead to death.

**Case presentation:**

We present a 44-year-old Caucasian female in whom cardiac tamponade manifested as venoarterial extracorporeal membrane oxygenation weaning failure. After discovering the contribution of cardiac tamponade, it was possible to wean the patient from venoarterial extracorporeal membrane oxygenation support. No clear signs of cardiac tamponade had existed beforehand.

**Conclusions:**

The diagnosis of cardiac tamponade can be very challenging in venoarterial extracorporeal membrane oxygenation supported patients due to (patho)physiological particularities related to the parallel blood flow.

**Supplementary Information:**

The online version contains supplementary material available at 10.1186/s13256-022-03741-9.

## Introduction

Cardiac tamponade can cause progressive and significant hemodynamic collapse [1]. To prevent circulatory arrest, timely recognition is essential. The diagnosis of cardiac tamponade is traditionally based on clinical and echocardiographic findings, which reflect the functional consequences of excessive fluid or thrombus in the pericardial sac [2].

In patients supported with venoarterial extracorporeal membrane oxygenation (VA ECMO) for cardiogenic shock, cardiac tamponade may manifest very differently. To our knowledge, the description of functional consequences of cardiac tamponade during VA ECMO support is very limited [3].

In this report, we illustrate the difficulties regarding the definite diagnosis and potentially detrimental consequences of cardiac tamponade in the setting of VA ECMO, and summarize the mechanisms by which VA ECMO may importantly mitigate classical clinical findings of cardiac tamponade.

## Case presentation

A 44-year-old Caucasian female with chronic obstructive pulmonary disease (COPD), diabetes, and hypothyroidism was admitted to a nonteaching hospital with severe biventricular cardiac failure *de novo*. The weeks before presentation, she experienced progressive exertional dyspnea and ankle edema. Echocardiography showed a severely reduced left ventricular ejection fraction (LVEF) (± 10%) with an apical thrombus. Her chest X-ray revealed severe pulmonary edema. For her dilating cardiomyopathy, no apparent cause was found and heart failure medical therapy [including diuretics, angiotensin-converting enzyme (ACE) inhibitors and low dosages of beta blockade] were initiated. Two days after admission, she developed a cardiac arrest due to ventricular fibrillation. Despite rapid return of spontaneous circulation following immediate advanced life support, cardiogenic shock persisted with high dosages of inotropes (dobutamine > 10 μg/kg/minute) and high levels of ventilator support [positive end expiratory pressure (PEEP) > 14 cm H_2_O) because of fulminant pulmonary edema.

In the absence of cardiosurgical facilities, she was transferred by a mobile intensive care unit and under inotropic support to our hospital. VA ECMO was initiated soon after arrival. Surgical cannulation (directly into the right atrium and central aorta after sternotomy) was chosen over a peripheral approach because of both local preferences and the presence of severe pulmonary dysfunction, anticipating for a significant risk for developing Harlequin syndrome. Additional unloading of the left ventricle (LV)  was effectuated by left ventricular venting via the right pulmonary vein and the pericardial sac was left open. Additional coronary angiography and myocardial biopsy did not reveal any treatable substrate. Within the following days, serial echocardiographic evaluation showed gradual biventricular improvement. Repeated weaning attempts were however unsuccessful because systemic hemodynamics deteriorated immediately after reduction of ECMO flow below 2 L/minute.

After 12 days of ECMO support, our patient still required norepinephrine 225 ng/kg/minute and milrinone 0.14 µg/kg/minute. She was in sinus rhythm (120/minute) with a mean arterial pressure of 68 mmHg, a central venous pressure (CVP) of 10 mmHg, and her SvO_2_ was 0.55. No pulsus paradoxus was present. As inflammatory parameters [C-reactive protein (CRP) 59 → 200 mg/L] vasopressor need increased, a suspicion of infectious complications of the central approach was feared. Therefore, it was decided to perform a resternotomy and convert from central to peripheral VA ECMO. In the presence of gradual improvement of myocardial contractility, extension of ECMO support was intended as a bridge to recovery since poor right ventricular (RV) function prohibited candidacy for long-term LV assist device therapy (LVAD). During surgery, a significant amount (300 mL) of pericardial fluid and thrombus mass was found and removed after which hemodynamics immediately improved, enabling subsequent VA ECMO weaning and decannulation during the same surgical procedure. Although the pericardium was left open, a large thrombus compressed the RV and caval veins. Remarkably, echocardiography just prior to sternotomy revealed some pericardial effusion but no clear thrombus or functional signs of tamponade (Additional files 1 and 2).

Four weeks after intensive care unit (ICU) admission, the patient could be transferred to the regular cardiology ward and at follow-up at 1 year after discharge, our patient was alive and asymptomatic with low dosages of ACE inhibitor and beta blockade therapy.

## Discussion

Our case report highlights the difficulties in diagnosing cardiac tamponade in the setting of VA ECMO, and its potential contribution to persistent failure to wean.

Cardiac tamponade has traditionally been considered a clinical diagnosis, where findings on physical examination and echocardiography reflect functional consequences of inflow obstruction due to pericardial effusion/thrombus. The pericardium’s inability to further distend at short notice and accommodate the increased blood flow to the right heart during inspiration leads to a shift of the interventricular septum towards the left ventricle. This septal shift further reduces LV preload leading to pulsus paradoxus [4]. During echocardiography, inflow obstruction is characterized by exaggeration of respiration-dependent transvalvular flow variations [5]. In addition, the visualization of a large thrombus mass with or without a clear impression on the right atrium and/or ventricle further underscores the suspicion of a cardiac tamponade.

**Fig. 1:**
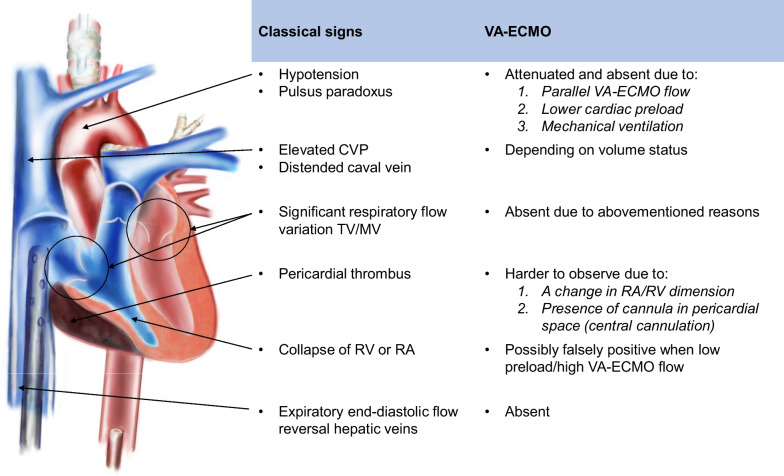
The hypothetical impact of VA ECMO on classical signs of cardiac tamponade

In our patient under VA ECMO support, cardiac tamponade did not produce any of the above mentioned characteristics. Importantly, in this context pathophysiological particularities as a consequence of VA ECMO interference needed to be considered as an explanation for the absence of these classical signs of cardiac tamponade (Fig. 1). First, as blood pressure may for a significant part depend on VA ECMO support paralleling the systemic circulation, hypotension or a trend towards lower blood pressure as a consequence of cardiac tamponade may become attenuated or even go unnoticed. For the same reason, a pulsus paradoxus and pathological respiratory changes in early diastolic transvalvular flow velocities are unlikely to arise. The absence of these patterns may further be masked by the effects of positive pressure mechanical ventilation [6]. Second, VA ECMO seems to affect anatomical landmarks and generally acknowledged echocardiographic references to an important extent.

A reduction in cardiac chamber dimensions that can occur during VA ECMO, be it with the adjunct use of LV unloading, can at times hardly be distinguished from a compression caused by a pericardial thrombus. The echogenicity may further be problematic when, in case of central cannulation, a cannula is positioned through the pericardial sac. Likewise, the position of the drainage cannula in the inferior caval vein also potentially compromises the proper evaluation of vascular dimensions and expiratory end-diastolic flow reversal in the hepatic vein.

Our case also demonstrated that the consequences of cardiac tamponade in the setting of VA ECMO seem to differ significantly as compared with those in patients without VA ECMO. Here, the only clue to its presence was suggested by persistent weaning failure despite gradual biventricular improvement. Whereas untreated cardiac tamponade in a nonsupported circulation is well known to cause sudden circulatory collapse, cardiac arrest, or death, it seems to impact circulation to a much lesser extent during VA ECMO support. Rather, cardiac tamponade during VA ECMO may present as (1) a very gradual deterioration in hemodynamics with only slowly increasing vasopressor dosages or (2) persistent failure to wean from VA ECMO. Both features could however also be secondary to many other causes. Dissecting this differential diagnosis during VA ECMO support is pivotal as cardiac tamponade can manifest as a reversible cause of persistent VA ECMO dependency. This seems especially relevant as indications for VA ECMO applications are broadening and its use is steadily growing [7].

## Conclusions

Our case illustrates that establishing a proper diagnosis of cardiac tamponade in the setting of VA ECMO support proves both cumbersome and critical. Daily management of VA ECMO should therefore include special consideration for a very distinct clinical manifestation of cardiac tamponade under VA ECMO, at times only becoming apparent as a persistent failure to wean from VA ECMO.

The classical signs of cardiac tamponade plus the presumed impact of VA ECMO on these signs are summarized and illustrated in Fig. 1. VA ECMO: venoarterial extracorporeal membrane oxygenation; CVP: central venous pressure; TV: tricuspid valve; MV: mitral valve; RA: right atrium; RV: right ventricle. The figure was drawn by one of the authors (C.L.M.).

## Supplementary Information

**Additional file 1.** Transgastric echocardiographical images of left and right ventricular function.

**Additional file 2.** Transoesophageal echocardiographical images of left and right ventricular function.

## Data Availability

Not applicable.
